# Membrane Type 1 Matrix Metalloproteinase induces an epithelial to mesenchymal transition and cancer stem cell-like properties in SCC9 cells

**DOI:** 10.1186/1471-2407-13-171

**Published:** 2013-04-01

**Authors:** Cong-Chong Yang, Li-Fang Zhu, Xiao-Hui Xu, Tian-Yun Ning, Jin-Hai Ye, Lai-Kui Liu

**Affiliations:** 1 Department of Basic Science of Stomatology, Institute of Stomatology, Nanjing Medical University, Nanjing, People’s Republic of China; 2 Department of Basic Science of Stomatology, College of Stomatology, Nanjing Medical University, Postal# 210029 136# Hanzhong Road, Nanjing, Jiangsu, People’s Republic of China; 3 Department of Stomatology, the First Affiliated Hospital of Soochow University, Suzhou, People’s Republic of China; 4 Department of the First Outpatient, College of Stomatology, Nanjing Medical University, Nanjing, People’s Republic of China; 5 Department of Oral and Maxillofacial Surgery, College of Stomatology, Nanjing Medical University, Nanjing, People’s Republic of China

**Keywords:** Membrane type 1 matrix metalloproteinase, EMT, Cancer stem cell, Oral squamous cell carcinoma

## Abstract

**Background:**

Tissue invasion and metastasis are acquired abilities of cancer and related to the death in oral squamous cell carcinoma (OSCC). Emerging observations indicate that the epithelial-to-mesenchymal transition (EMT) is associated with tumor progression and the generation of cells with cancer stem cells (CSCs) properties. Membrane Type 1 Matrix Metalloproteinase (MT1-MMP) is a cell surface proteinase, which is involved in degrading extracellular matrix components that can promote tumor invasion and cell migration.

**Methods:**

In the current study, we utilized SCC9 cells stably transfected with an empty vector (SCC9-N) or a vector encoding human MT1-MMP (SCC9-M) to study the role of MT1-MMP in EMT development.

**Results:**

Upon up-regulation of MT1-MMP, SCC9-M cells underwent EMT, in which they presented a fibroblast-like phenotype and had a decreased expression of epithelial markers (E-cadherin, cytokeratin18 and β-catenin) and an increased expression of mesenchymal markers (vimentin and fibronectin). We further demonstrated that MT1-MMP-induced morphologic changes increased the level of Twist and ZEB, and were dependent on repressing the transcription of E-cadherin. These activities resulted in low adhesive, high invasive abilities of the SCC9-M cells. Furthermore, MT1-MMP-induced transformed cells exhibited cancer stem cell (CSC)-like characteristics, such as low proliferation, self-renewal ability, resistance to chemotherapeutic drugs and apoptosis, and expression of CSCs surface markers.

**Conclusions:**

In conclusion, our study indicates that overexpression of MT1-MMP induces EMT and results in the acquisition of CSC-like properties in SCC9 cells. Our growing understanding of the mechanism regulating EMT may provide new targets against invasion and metastasis in OSCC.

## Background

Oral squamous cell carcinoma (OSCC) is a major oral cavity health problem. Although many therapeutic strategies have been carried out [1], the 5-year survival rate for these patients has remained at 50–60% for the last three decades [2]. Tissue invasion and metastasis are exceedingly complex processes and are one of the hallmarks of cancer [3]; thus, it is important to clarify the biological mechanism of tissue invasion and metastasis for grading the course of cancer and developing more effective therapies [3,4].

The epithelial-to-mesenchymal transition (EMT) is the cellular and molecular process through which cell-to-cell interactions and apico-basal polarity are lost and a mesenchymal phenotype is acquired, which are required for cell motility and basement membrane invasion during metastasis [5,6]. The EMT plays a critical role in embryogenesis and is associated with tissue remolding, wound healing, fibrosis, cancer progression and metastasis [5,7-9]. In the metastatic cascade of epithelial tumors, the EMT has been established as an important step [10]. Furthermore, researchers have shown that the EMT is associated with the dedifferentiation program that leads to malignant carcinoma [5], as the EMT confers invasive cancer cells an efficient migration ability and a selective advantage to reach distant locations [9,10]. Transcriptional repression of the E-cadherin gene can lead to the loss of the epithelial phenotype and the functional loss of E-cadherin is one of the hallmarks of EMT [5]. In particular, transcriptional repressor has recently emerged as a fundamental mechanism for the silencing of CDH1 (the gene that encodes E-cadherin), such as the Snail (Snail1 and Slug), ZEB (ZEB1 and ZEB2) and basic helix-loop-helix (bHLH: Twist) families [6,11].

Matrix metalloproteinases (MMPs) are zinc-dependent endopeptidases. MMPs are involved in degrading extracellular matrix (ECM) in normal physiological processes, such as embryonic development, reproduction and tissue remodeling, as well as in disease processes, such as arthritis and metastasis [12,13]. There are over 23 MMPs identified in humans, which are subdivided into soluble MMPs and membrane-type MMPs (MT-MMPs) [14,15]. While MT1-MMP has a common MMP domain structure with a signal peptide, a pro-peptide, catalytic and hemopexin-like domains, it also has unique insertions. One of the insertions is at the C-terminus and contains a hydrophobic amino-acid sequence that acts as a transmembrane domain [16,17]. As a member of the MMPs, MT1-MMP is closely associated with cancer invasiveness and the promotion of cell migration [16,18-20]. Recent researches have emerged to indicate that cell surface MT1-MMP has been recognized as an inducer of EMT in cancer cells [21,22]. The researches on MT1-MMP further demonstrated that MT1-MMP via cleaving E-cadherin induced an EMT in transfected breast cancer [21], which was shown to be dependent on up-regulation of Wnt5a in prostate cancer cells [22]. However, the molecular transcriptional mechanism related to MT1-MMP as an inducer of EMT remains poorly understood, and the association of MT1-MMP and EMT has not been reported in oral cancer cells. Thus, we examined whether MT1-MMP-induced EMT through mediation of transcriptional repression of E-cadherin in OSCC.

Recently, studies of neoplastic tissues have provided evidence of self-renewing, stem-like cells within tumors, which have been called cancer stem cells (CSCs) [23]. Increasing evidence suggests that EMT bestows carcinoma cells at the tumor front with cancer stem cell (CSC)-like properties and plays an important role in initiating CSCs [24,25]. Furthermore, CSCs have been identified in head and neck SCC [4,25]. However, an association specifying the EMT and CSCs induced by MT1-MMP in SCC9 cells has not been investigated.

Based on the above studies, we demonstrate the molecular mechanisms in OSCC that are involved in the overexpression of MT1-MMP by the cancer cells that induces an EMT and leads to the acquisition of CSC-like properties by the cancer cells. These studies may provide new avenues of research with potential clinical implications.

## Methods

### Cell cultrue, plasmid construction and transfection

Human oral squamous cell carcinoma SCC9 cells were obtained from the American Type Culture Collection (ATCC, Manassas, VA, USA). Cells were maintained in a mixture of Dulbecco’s Modified Eagle’s medium and Ham’s F12 medium (1:1) (Invitrogen, Burlington, Ontario, Canada) supplemented with 10% fetal bovine serum (FBS, Invitrogen), 400 ng/ml hydrocortisone (Sigma-Aldrich, St Louis, MO, USA) and penicillin (100 U/ml)/streptomycin (100 μg/ml) (Invitrogen). A full-length cDNA for human MT1-MMP (NM_004995.2) was amplified using RT-PCR and then ligated into the PCR2.1-TOPO vector. The constructed PCR gene product was cloned into the pEGFP-N1 vector. The final gene synthesis was verified via sequencing and amplified using DH5α competent cells. The Endo-free Plasmid Mini Kit II (OMEGA) was used for all plasmid preparations. For transfection experiments, cells were maintained in six-well plates (Corning, Lowell, MA, USA) and cultured to 80% confluence, after which the medium was changed to serum-free medium for overnight incubation. The cells were transfected with Lipofectamine 2000 (Invitrogen) according to the manufacturer’s instructions. G418 (400 μg/ml; Invitrogen) was added to the media 48 h after transfection. The cells were allowed to grow in the presence of G418 for two weeks, and clones were picked for growth on plates to confluence. Thus, stably expressing empty vector--SCC9-pEGFP-N cells (SCC9-N) and a vector encoding human MT1-MMP--SCC9-pEGFP-M cells (SCC9-M) were obtained for our study.

For the experiment of addition of inhibitors, 2×10^5^/ml SCC9-M cells were added to six-well plates (Corning). The cells were then treated with 5 nM tissue inhibitor of metalloproteinase (TIMP)-1 (Calbiochem, Darmstadt, Germany), 5 nM of TIMP2 (Calbiochem) and incubated for three days at 37°C.

### Real-time RT-PCR

Total RNA was extracted from cells using the TRIzol reagent (Invitrogen). For cDNA synthesis, mRNA was reverse-transcribed into cDNA using the 5×PrimeScript RT Master Mix (TaKaRa) at 37°C for 15 min and 85°C for 5 s according to the manufacturer’s protocol. Gene expression was quantified by real-time quantitative PCR using 2×SYBR Premix Ex Taq (TaKaRa) with a 7300 ABI Real-Time PCR System (Applied Biosystems, Foster City, CA, USA) under the conditions of 95°C for 30 s, 95°C for 5 s, and 60°C for 31 s for 40 cycles. The relative gene expression was calculated using the 2(−ΔΔCT) method. Briefly, the resultant mRNA was normalized to its own GAPDH [26]. The following primers were utilized for the real-time RT-PCR. GAPDH (5^′^-GAAGGTGAAGGTCGGAGTC-3^′^, 5^′^-GAGATGGTGATGGGATTTC -3^′^), MT1-MMP (5^′^-GGAACCCTGTAGCTTTGTGTCTGTC-3^′^, 5^′^-TGAGGGTCCTGCCTTCAAGTG-3^′^), E-cadherin (5^′^-TACACTGCCCAGGAGCCAGA-3^′^, 5^′^-TGGCACCAGTGTCCGGATTA-3^′^), β-catenin (5^′^-GCTGAAGGTGCTATCTGTCTGCTC-3^′^, 5^′^-TGAACAAGACGTTGACTTGGATCTG-3^′^), cytokeratin18 (5^′^-AGGAGTATGAGGCCCTGCTGAA-3^′^, 5^′^-TTGCATGGAGTTGCTGCTGTC-3^′^), vimentin (5^′^-TGAGTACCGGAGACAGGTGCAG-3^′^, 5^′^-TAGCAGCTTCAACGGCAAAGTTC-3^′^), fibronectin (5^′^ –TGCCTTGCACGATGATATGGA-3^′^, 5^′^-CTTGTGGGTGTGACCTGAGTGAA-3^′^), snail (5^′^-GACCACTATGCCGCGCTCTT-3^′^, 5^′^-TCGCTGTAGTTAGGCTTCCGATT-3^′^), slug (5^′^-ATGCATATTCGGACCCACACATTAC-3^′^, 5^′^-AGATTTGACCTGTCTGCAAATGCTC-3^′^), Twist (5^′^-GGAGTCCGCAGTCTTACGAG-3^′^, 5^′^-TCTGGAGGACCTGGTAGAGG-3^′^), ZEB1 (5^′^-GAAAGTGATCCAGCCAAATGGAA-3^′^, 5^′^-TTTGGGCGGTGTAGAATCAGAG-3^′^), ZEB2 (5^′^-AAATGCACAGAGTGTGGCAAGG-3^′^, 5^′^-CTGCTGATGTGCGAACTGTAGGA-3^′^) and CDH1 (5^′^-AGATGGTGTGATTACAGTCAAAAGG-3^′^, 5^′^-CAGGCGTAGACCAAGAAAT-3^′^).

### Western blotting and shedding of the E-cadherin ectodomain

Cells were lysed using a RIPA lysis buffer (Beyotime). Total protein (30 μg) from each sample was subjected to the SDS-PAGE and then transferred to PVDF membranes (Millipore, Billerica, MA, USA), which were blocked for 2 h at room temperature with 5% nonfat milk in PBST. The following antibodies were used to detect bands on the protein blots: anti-β-actin (1:1000, Santa Cruz Biotechnology, Santa Cruz, CA, USA), anti-MT1-MMP (1:500, Abcam, Cambridge, MA, USA), anti-E-cadherin (1:1000, Cell Signaling Technology, Danvers, MA, USA), anti-β-catenin (1:500, Santa Cruz Biotechnology), anti-cytokeratin18 (1:500, Bioworld Technology, MN, USA), anti-vimentin (1:500, Santa Cruz Biotechnology), anti-fibronectin (1:500, Santa Cruz Biotechnology), anti-Snail (1:500, Abcam), anti-Slug (1;1000, Cell Signaling Technology), anti-Twist (1:500, Abcam), anti-ZEB1 (1:300, Abcam) and anti-ZEB2 (1:500, Novus Biologicals, Littleton, USA). Immunoreactive material was visualized using the Immun-Star WesternC Kit (Bio-Rad, Hercules, CA, USA) products and bands were detected via exposure to film (Kodak, Japan). For detecting the expression of extracellular E-cadherin, the cells were cultured with serum-free medium for 24 h. Next, the conditioned medium was collected via centrifugation and concentrated 10-fold with a VirTis freeze dryer (SP Scientific, NY, USA). An immunoblot was performed as described above using an anti-E-cadherin ectodomain antibody (1:500, Santa Cruz Biotechnology). All western bolt analyses were performed at least three independent experiments.

### Immunofluorescence

Cells were cultured on glass coverslips, fixed in 4% paraformaldehyde (PFA) for 20 min at room temperature, permeabilized with 1% Triton X-100 for 15 min and blocked with goat serum albumin for 30 min 37°C, followed by an overnight incubation at 4°C with antibodies specific for E-cadherin (1:100, Cell Signaling Technology) and vimentin (1:100, Santa Cruz Biotechnology), or cytokeratin 18 (1:100, Bioworld technology) and fibronectin (1:100, Santa Cruz Biotechnology). The appropriate secondary antibodies (diluted 1:50) were then used, and then nuclei were stained by 4, 6-diamidino-2-phenylindole (DAPI; 1:1000, Invitrogen) for 2 min. Immunofluorescence was visualized using a Zeiss LSM-710 laser-scanning confocal microscopy.

### Adhesion, invasion and wound healing assays

The cells were plated in six-well plates (Corning) at a density of 4×10^5^ per well and then trypsinized after 1 and 2 h, respectively. The attached cells were counted under an inverted microscope (Olympus), and the adherent rate of the three different cell populations was calculated. The cell invasion was assessed using Transwell filters with 6.5-mm diameters and 8-μM pore sizes (Costar, Lowell, MA, USA). The filters were precoated for 30 min at 37°C with 50 μL per square centimeter of growth surface with Matrigel Basement Membrane Matrix (BD Biosciences, MA, USA) diluted with serum-free medium (1:3) according to the manufacturer’s procedures. The cells (3×10^5^) were resuspended with 100 μl serum-free medium inoculated in the upper chamber while 500 μl medium containing 10% FBS was placed in the lower chamber. The plates were placed at 37°C in 5% CO_2_ for 24 h. The chambers were fixed with 4% PFA and stained with 0.1% crystal violet (Beyotime) for 30 min. The non-migratory cells were removed, and the migratory cells were counted as those presenting on the lower surface of the upper chamber. Images of at least ten random fields per chamber were captured (×100 magnification). For the wound healing assay, the cells were allowed to grow to 90% confluence and then wounded by scratching with a pipette tip in the central area. Floating cells and debris were removed, and the medium was changed to serum-free. The cells were incubated for 48 h to allow cells to grow and close the wound. Photographs were taken at the same position of the wound at the indicated time points.

### Flow cytometry

For flow cytometric cell-cycle analysis, the cells were synchronized with serum-free medium for 24 h, released and then cultured for three days. The cells were detached from the culture plate with trypsin, washed with PBS, and then resuspended in 75% alcohol. The prepared cells were stained with 100 mg/ml of propidium iodide (BD Pharmingen, San Jose, CA, USA) prior to analysis using flow cytometry with a BD FACS Calibur (BD Biosciences) and CellQuest Pro software (BD Biosciences). For surface marker analysis, the cells were collected and then labeled with human-fluorochrome-conjugated anti-CD24-PE (10 μl per test, Beckman Coulter, Los Angeles, CA, USA), anti-CD44-APC (20 μl per test, BD Pharmingen), anti-CD133-PE (10 μl per test, Miltenyi Biotech, Auburn, CA, USA). The corresponding mouse immunoglobulins conjugated to PE or APC (BD Pharmingen) were used as isotype controls in each experiment. For apoptosis analysis, the cells were dealed with mitomycin at concentration gradients of 16 and 128 mg/ml for 24 h. Then the prepared cells were collected and stained with PE Annexin V Apoptosis Detection Kit I (BD Pharmingen) for 15 min according to the manufacturer’s protocol. The rate of apoptosis cells was relative to each untreated group.

### Colony-forming assays

The cells were plated in 100-mm dishes (Corning) at a density of 1000 cells per dish and cultured at 37°C for two weeks. The dishes were fixed in 4% PFA, stained with crystal violet, and photographed. The colonies were visualized under an inverted microscope (Olympus). Aggregations of more than 50 cells were defined as a colony.

### MTT assay

The survival rate of cells was analyzed using an MTT (Sigma) assay, which is a colorimetric assay for measuring the activity of enzymes that reduce MTT to formazan dyes, producing a purple color. The MTT assay is the preferred method used to assess the viability and proliferation of cells [27]. The SCC9-N and SCC9-M cells were plated in 96-well plates (Corning) at an initial density of 2×10^3^ cells per well, and then synchronized with serum-free medium for 24 h. For consecutive culturing at 0, 1, 3, 5, 7, 9 d, the cells were treated with 5 mg/ml MTT and incubated at 37°C for 4 h, and then treated by dimethylsulfoxide (Sigma). The absorbance of samples in triplicate wells was measured with an automatic enzyme-linked immunosorbent assay reader (ELx800, BioTek Instruments, Inc., USA) at a wavelength of 490 nm. Population doubling time (PDT) was calculated according to Patterson formulation. For drug resistant experiment, the SCC9-N and SCC9-M cells were plated in 96-well plates (Corning) at the same density of 5×10^4^ cells. After serum-starvation, mitomycin at concentration gradients of 16 and 128 mg/ml was added separately to the culture medium and maintained for 24 h. The absorbance of samples in triplicate wells was measured as introduced above. The survival rate of the cells relative to each untreated group was calculated. The data were analyzed using three independent experiments.

### Statistical analysis

The data were representative of three or more independent experiments as the mean ± s.d. Statistical significance was assessed using one-way analysis of variance and Student’s unpaired t test. P-value <0.05 was considered significant.

## Results

Human oral squamous cell carcinoma SCC9 cells are less aggressive, which may correlate with the low MT1-MMP expression level observed in these cells. Thus, we utilized the up-regulation of MT1-MMP in SCC9 cells, via the transfection of either an empty vector (SCC9-N) or a vector encoding human MT1-MMP (SCC9-M), to study the role of MT1-MMP in cancer invasion and metastasis. After screening via G418 selection, we performed a series of experiments using SCC9 cell lines stably expressing empty vector or MT1-MMP.

### MT1-MMP induces SCC9 cells to undergo an EMT and alters cell phenotype

**Figure 1:**
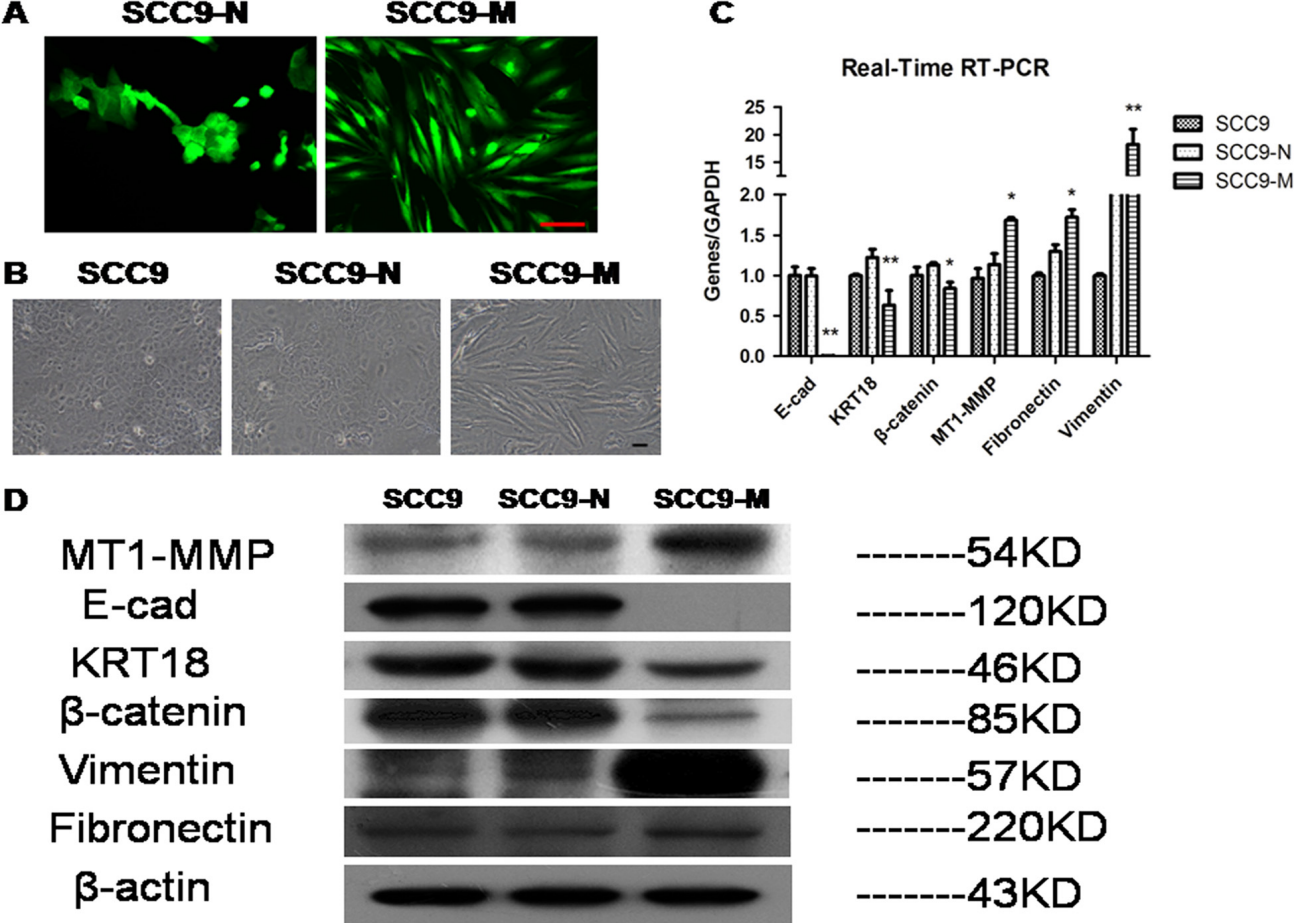
**MT1-MMP induces SCC9 cells to undergo an EMT and morphologic changes.** (**A**) Stable expression of an empty vector or MT1-MMP in SCC9 cells was established. Pictures were captured under fluorescence microscope. Bar, 100 μm. (**B**) Stable SCC9 cells expressing empty control vector (SCC9-N) possessed a typical epithelial phenotype, similar to parental SCC9 cells. Stable SCC9 cells expressing MT1-MMP (SCC9-M) possessed an elongated, fibroblastic appearance. Photographs were taken under inverted microscope (Olympus). Bar, 100 μm. (**C**) Quantitative determination of mRNA expression of epithelial markers (E-cadherin, cytokeratin 18 and β–catenin) and mesenchymal markers (vimentin and fibronectin) in SCC9, SCC9-N, SCC9-M cells using real-time RT-PCR. GAPDH was used as a control. Each bar represents the mean ± s.d. *P<0.05, **P<0.01. (**D**) The EMT-related protein levels were characterized by Western blot analysis. β-actin was employed as a loading control.

**Figure 2:**
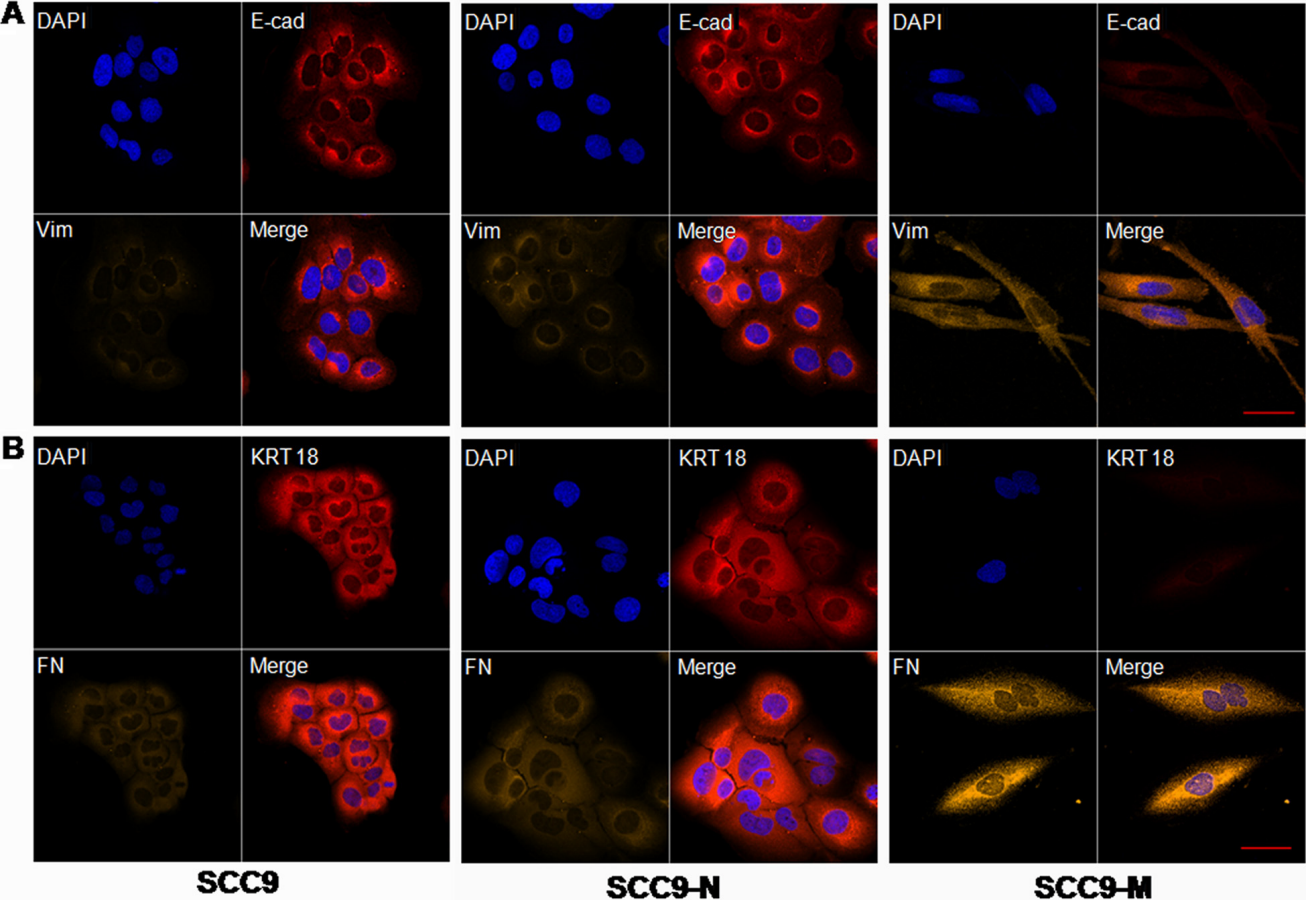
**Immunofluorescence analysis of SCC9 cells, stable SCC9 cells expressing an empty vector (SCC9-N) and MT1-MMP (SCC9-M).** (**A**) Double immunofluorescence staining of E-cadherin (E-cad: red) and vimentin (Vim: orange). (**B**) Double immunofluorescence staining of cytokeratin 18 (KRT 18: red) and fibronectin (FN: orange). The nuclei in both image sets were stained with DAPI (blue). Images were taken at ×400 magnification. Bar, 100 μm.

Overexpression of MT1-MMP in SCC9 cells results in morphologic changes of cells that are undergoing an obvious EMT. The observed morphological changes observed include cells switching from a cuboid epithelial shape to a fibroblastic appearance, which was not observed in cells expressing a control empty vector. The same changes can be viewed under a fluorescence microscope (Figure 1A and B). The quantitative determination of the mRNA expression in SCC9-M cells using real-time RT-PCR revealed that a loss of the epithelial markers E-cadherin, cytokeratin 18 and β–catenin occurred when compared with that of GAPDH. Simultaneously, an up-regulation of the mensenchymal markers vimentin and fibronectin was observed (Figure 1C). To verify these findings on the protein level, a Western blot analysis was utilized to determine the expression of the epithelial and mesenchymal markers. Notably, the Western blot analysis confirmed the changes in gene expression, as the increased expression of MT1-MMP in SCC9 cells resulted in a decrease of E-cadherin, cytokeratin 18, and β–catenin, and a concurrent increase of vimentin and fibronectin (Figure 1D). To confirm the effect of MT1-MMP on the EMT of the cells, we used laser scanning confocal fluorescence microscopy to identify the expression of these epithelial and mesenchymal markers. As shown with the immunofluorescence analysis, the SCC9-M cells presented a fibroblast-like appearance, with decreased E-cadherin (red) and cytokeratin 18 (red) and increased vimentin (orange) and fibronectin (orange) protein expression. By contrast, the SCC9-N cells retained an epithelial-like morphology with normal E-cadherin and cytokeratin 18 expression and weak vimentin and fibronectin expression, similar to parental SCC9 cells (Figure 2A and B). These results demonstrated that increased MT1-MMP expression was capable of inducing an EMT in SCC9 cells.

### MT1-MMP induces EMT is associated with an increase of Twist and ZEB and through repressing the transcription of E-cadherin

**Figure 3:**
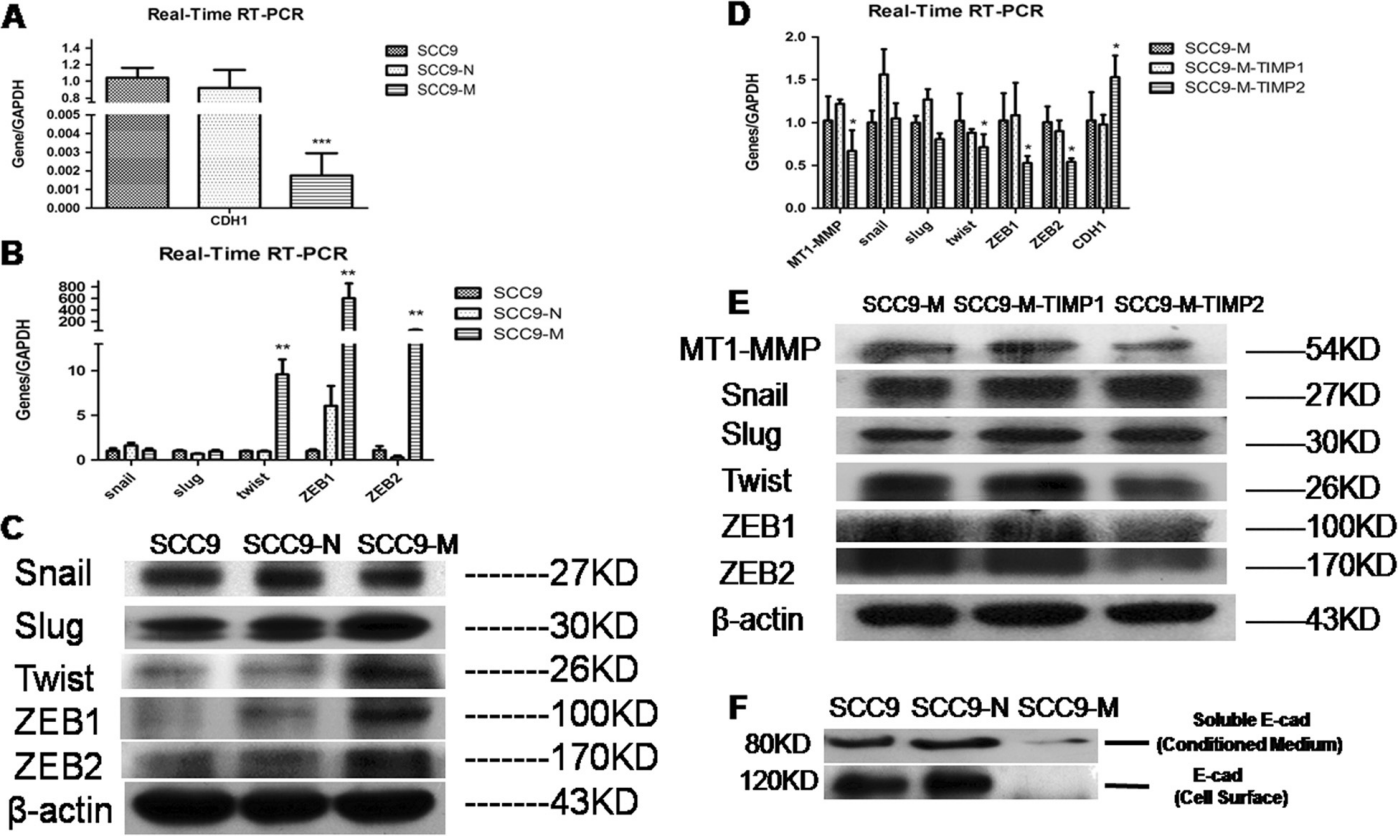
**MT1-MMP induces SCC9 cells to undergo EMT is associated with an increased level of Twist and ZEB and through repressing the transcription of E-cadherin.** (**A**) Real-time RT-PCR was performed to detect the expression of CDH1 that encoding E-cadherin in SCC9 cells, stable SCC9 cells expressing an empty vector (SCC9-N) and MT1-MMP (SCC9-M). GAPDH was used as a control. Each bar represents the mean ± s.d. ***P<0.001. (**B**) Real-time RT-PCR was performed to detect the key transcription factors that repressed CDH1 as inducers of EMT, including Snail, Slug, Twist, ZEB1 and ZEB2. GAPDH was used as a control. Each bar represents the mean ± s.d. **P<0.01. (**C**) Western blot was performed to analyze the expression of transcriptional repression factors on the protein level. β-actin was employed as a loading control. (**D** and **E**) Quantitative determination of mRNA expression of transcription factors on the SCC9-M cells treated with TIMP1 and TIMP2. Each bar represents the mean ± s.d. *P<0.05. The western blot analysis was performed to assess the expression of transcription factors on the SCC9-M cells treated with TIMP1 and TIMP2 on the protein level. β-actin was employed as a loading control. (**F**) The examination of the shedding of E-cadherin extracellular domain in conditioned medium and cell surface of SCC9, SCC9-N and SCC9-M.

The loss of functional E-cadherin is a hallmark of EMT [5] and is considered a prerequisite. To investigate the mechanism by which MT1-MMP induces SCC9 cells to undergo EMT, we detected the gene expression of CDH1 that encodes E-cadherin in SCC9, SCC9-N and SCC9-M cells, and also detected the expression of key transcriptional repressors of CDH1 as inducers of EMT. The result of real-time RT-PCR showed that the level of CDH1 was decreased to 0.0018-fold in SCC9-M cells relative to the SCC9 and SCC9-N cells (Figure 3A). Elevated levels of the mRNA for three key EMT-inducing transcription factors, Twist (9.55-fold), ZEB family-ZEB1 (602.03-fold) and ZEB2 (49.79-fold), were observed in SCC9-M cells relative to the SCC9 and SCC9-N cells. However, there was no significant difference in mRNA expression in Snail family members (Snail and Slug), as determined by real-time RT-PCR in the three cell lines (Figure 3B). Next, we proceeded to analyze the expression of these transcriptional repressors at the protein level. The Western blot trends corresponded to the real-time RT-PCR results (Figure 3C). A reduction of Twist (28.61%), ZEB1 (47.18%) and ZEB2 (45.92%) could be observed with SCC9-M cells in the presence of recombinant TIMP2 (5 nM, an inhibitor of MT1-MMP) but not TIMP1 (5 nM, an MMP inhibitor that does not specific affect MT1-MMP). The expression of CDH1 in SCC9-M cells was increased 52.33% by the addition of TIMP2 but not TIMP1 (Figure 3D and E). These results suggested that an MT1-MMP directed the process that regulating the expression of Twsit, ZEB and CDH1. Furthermore, the examination of the shedding of E-cadherin extracellular domains in conditioned medium was nearly undetected in SCC9-M cells (Figure 3F). These data indicated that the MT1-MMP-induced EMT was associated with an increased level of Twist and ZEB family and was dependent on repressing the transcription of E-cadherin.

### Overexpression of MT1-MMP in SCC9 cells results in a change in the biological properties of the cells

**Figure 4:**
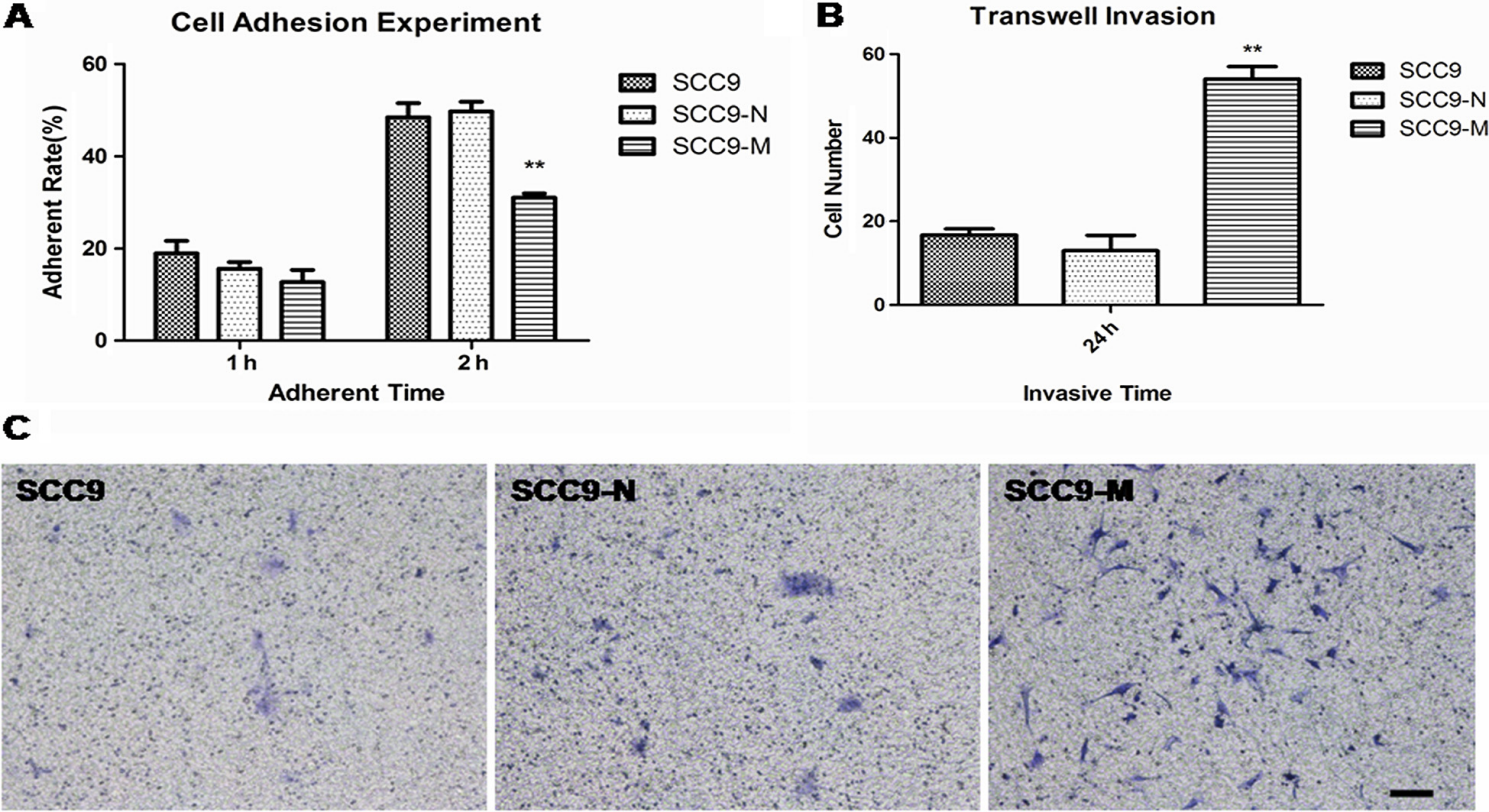
**The examination of the biological properties of SCC9 cells, stable SCC9 cells expressing an empty vector (SCC9-N) and MT1-MMP (SCC9-M).** Overexpression of MT1-MMP in SCC9 cells resulted in a low adhesive, high invasive ability. (**A**) The quantification of cell adhesion rate at two time points. Each bar represents the mean ± s.d. **P<0.01. (**B**) Quantitative analysis of cell invasion in ten different random fields. The data were calculated as the mean ± s.d. **P<0.01. (**C**) Pictures presenting the cells penetrating the Matrigel basement membrane matrix after 24 h. Bar, 100 μm.

**Figure 5:**
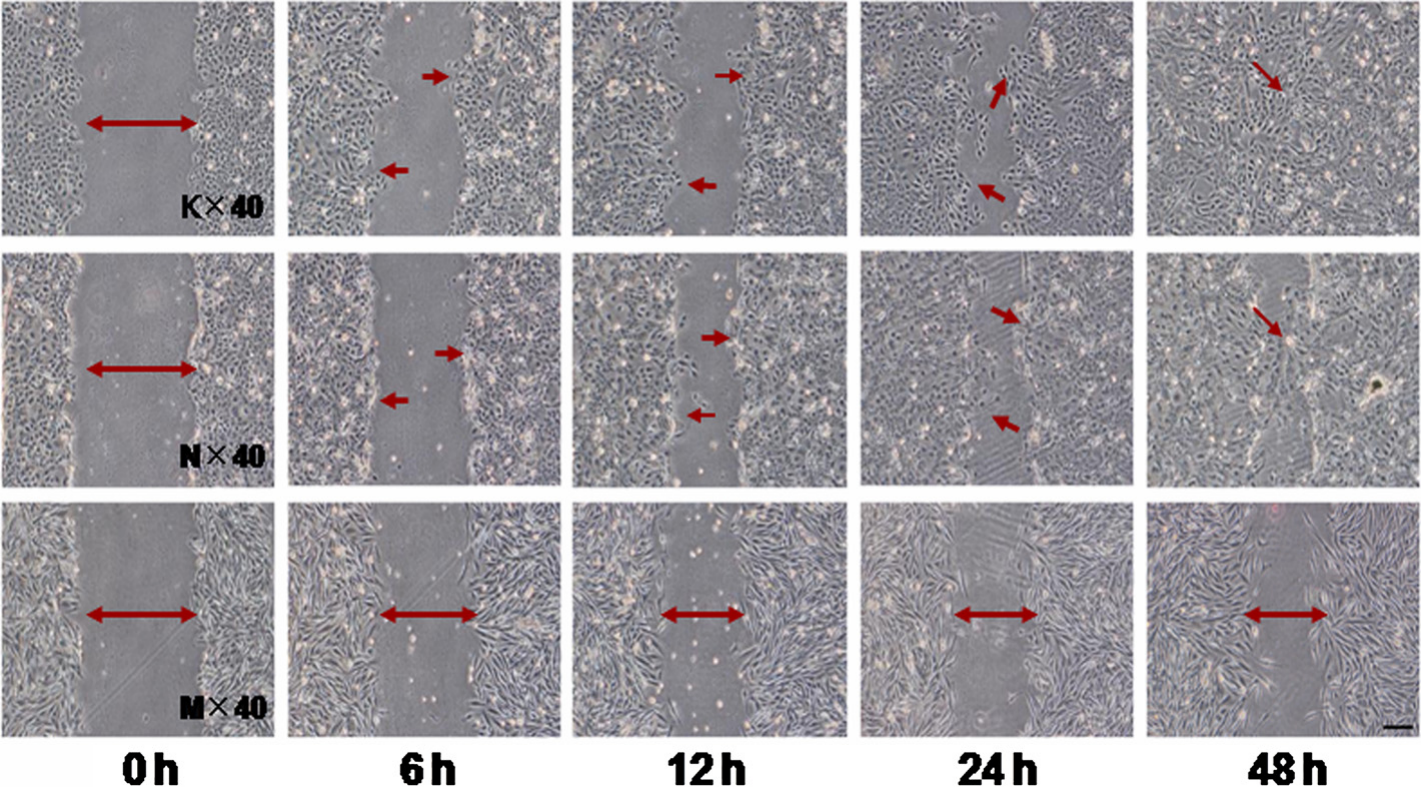
**The wound healing assay indicated that SCC9 cells transfected with MT1-MMP (SCC9-M) did not have the ability to close the wound after 48 h.** Photographs were taken at the same position of the wound at the indicated time points (×40 magnification). Bar, 100 μm. K, SCC9 cells. N, stable SCC9 cells expressing empty vector. M, stable SCC9 cells expressing vector encoding MT1-MMP.

In previous studies, the mesenchymal cells were highly invasive and metastatic, with a loss of cell-to-cell adhesion [28]. To identify whether MT1-MMP induces this ability of SCC9 cells to gain the mesenchymal-like appearances with these characteristics, we performed a series of experiments. First an adhesion test demonstrated that the SCC9-M cells had a lower adhesive ability than the SCC9 and SCC9-N cells (Figure 4A). The adherent rate of the cells at one hour was 18.95%, 15.63% and 12.71% for the SCC9, SCC9-N and SCC9-M cells, respectively. At two hours, the percentage of attached cells for the three cell lines was 48.46%, 49.79%, and 31.04%, respectively. Next, a Transwell assay was performed to evaluate the invasive ability of SCC9-M cells. After 24 h, the ability of the cells to penetrate Matrigel basement membrane matrix was quantified and captured with ×100 magnification in ten random fields. The photographs demonstrated that the SCC9-M cells were more invasive than SCC9 and SCC9-N cells. The quantitative analysis revealed an increase of 2.84-fold or 3.38-fold over that observed for SCC9 or SCC9-N cells (Figure 4B and C). These results demonstrated that increased expression of MT1-MMP promoted the invasive ability of SCC9 cells. The result of scratch test showed that the SCC9 and SCC9-N cells migrated to confluence after 48 h; however, the SCC9-M cells exhibited no ability to close the wound (Figure 5). Previous study suggested that wound-healing assays had been carried out in tissue culture for many years to estimate the proliferation rates and migratory behavior associated with different cells and culture conditions [29]. This result illuminated that the more invasive SCC9-M cells presented to a low growth ability, which was correlated with the phenomenon observed during cell culture. Previous study showed that there existed a subpopulation of tumor cells with stem cell-like characteristics such as very slow replication and resistance to chemotherapy [30]. Thus, we speculated that overexpression of MT1-MMP in SCC9 cells resulted in the cells undergoing an EMT and presented lower proliferation ability that may confer CSC-like properties.

### Overexpression of MT1-MMP in oral cancer cells results in the expression of CSC-like characteristics

**Figure 6:**
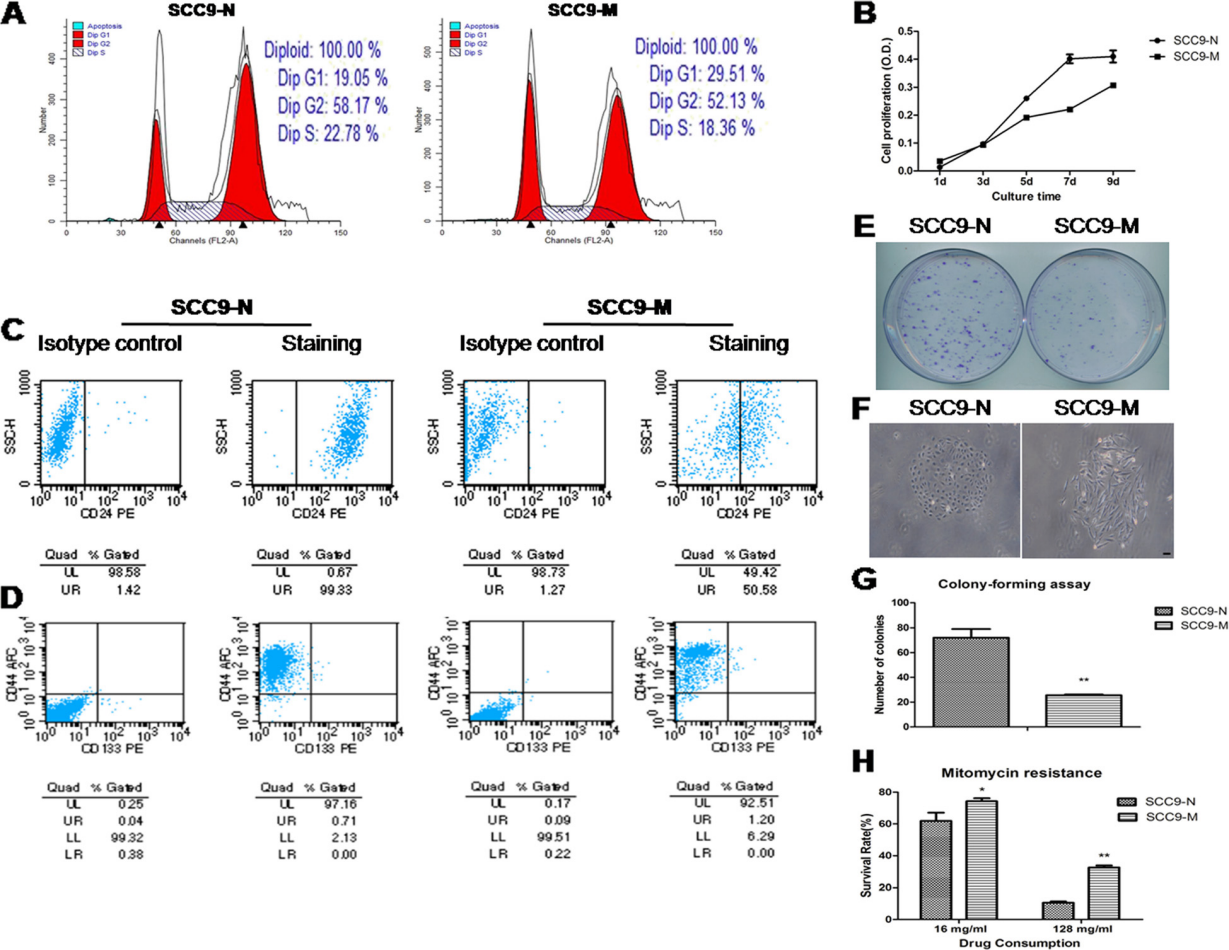
**Stable SCC9 cells expressing MT1-MMP (SCC9-M) presented cancer stem cell (CSC)-like properties of expressing CSC markers, self-renewal and resistance to drugs.** (**A**) Cell-cycle analysis of stable SCC9 cells expressing empty vector (SCC9-N) and MT1-MMP (SCC9-M). (**B**) Growth curves of SCC9-N and SCC9-M cells. The bar represents the mean ± s.d. (**C** and **D**) The flow cytometry analysis of CSC markers, including the CD24, CD44, and CD133 expression, in the SCC9-N and SCC9-M cells. The corresponding mouse immunoglobulins conjugated to PE or APC were used as isotype controls in each experiment. (**E**) Images of colonies stained with crystal violet for the SCC9-N and SCC9-M cells. (**F**) The pictures of cells forming single colony under microscopy. The photographs were taken at ×40 magnification. Bar, 100 μm. (**G**) Quantification of colonies formed by the SCC9-N and SCC9-M cells. The bar represents the mean ± s.d. **P<0.01. (**H**) The survival rate of SCC9-N and SCC9-M cells by the treatment with mitomycin after 24 h. Each bar represents the mean ± s.d. *P<0.05, **P<0.01.

**Figure 7:**
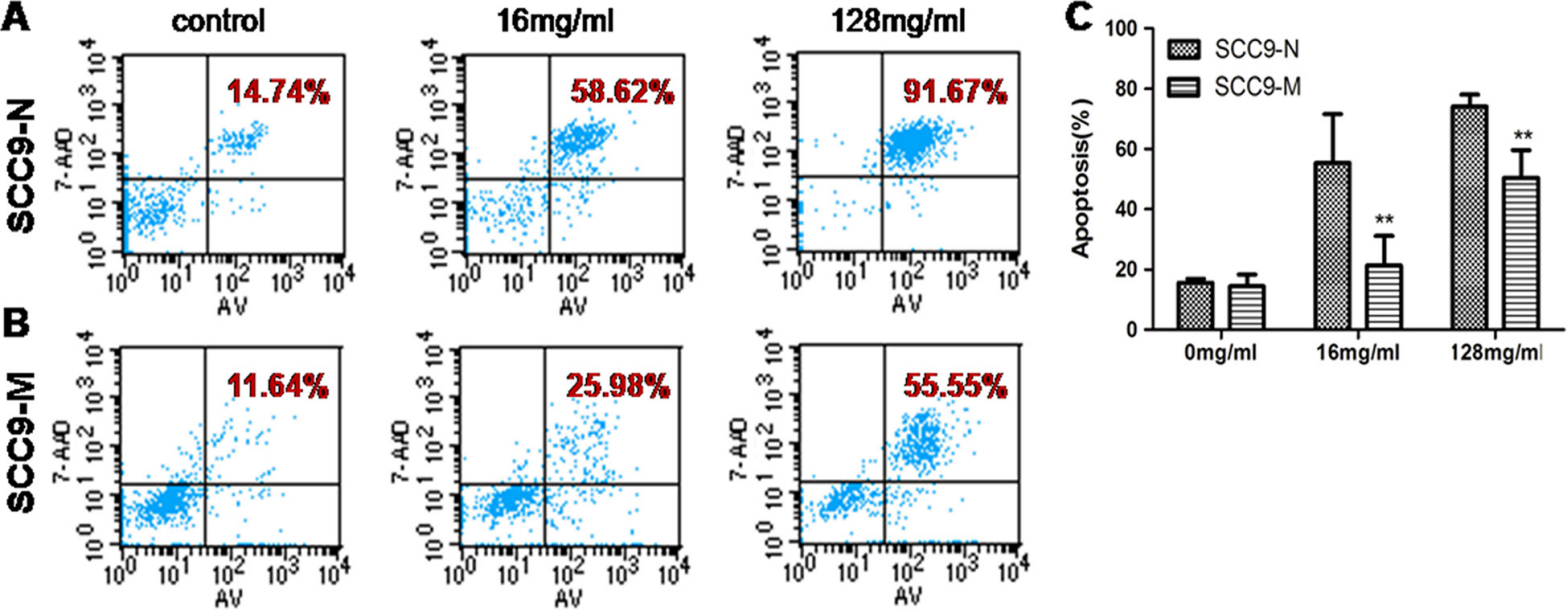
**Stable SCC9 cells expressing MT1-MMP (SCC9-M) possessed the ability of more resistant to apoptosis.** (**A** and **B**) The flow cytometric apoptosis analysis for stable SCC9 cells expressing an empty vector (SCC9-N) and SCC9-M cells under two concentrations of mitomycin. (**C**) The statistic analysis of the rate of cell apoptosis for the SCC9-N and SCC9-M cells. Each bar represents the mean ± s.d. **P<0.01.

Head and neck squamous cell carcinoma (HNSCC) contain a subpopulation of cancer cells that are capable of self-renewal, are able to proliferate and form new tumors, and possess the features of CSCs [4]. Previous studies on CSCs revealed that CSCs retain the properties of relative quiescence as well as resistance to therapeutic drugs and apoptosis [31,32]. To verify that SCC9-M cells had low proliferation abilities as shown in the scratch test, a cell-cycle analysis was performed. The SCC9-M cells had a higher percentage of cells in the G0/G1 phase (29.51%) than that observed for the SCC9-N cells (19.05%). In contrast, the total percentage of cells in S-phase was 18.36% for the SCC9-M cells, which is lower than the 22.78% observed for the SCC9-N cells, confirming that the SCC9-M displayed decreased proliferation ability (Figure 6A). An MTT assay was performed to further determine that the SCC9-M cells displayed a lower cell proliferation. As shown in cell growth curve, the PDT in SCC9-M cells (46.38 ± 1.14 h) was significantly longer than in SCC9-N cells (29.36 ± 1.35 h) (Figure 6B). Next we examined the expression levels of several CSCs surface markers by flow cytometry. The SCC9-M cells presented as CD44+ (93.45%) CD24-low (49.21%) CD133- (0.89%), while SCC9-N cells presented as CD44+ (97.58%) CD24-high (97.91%) CD133- (0.29%) (Figure 6C and D). A colony-forming assay was performed and confirmed that the SCC9-M cells had the ability to self-renew but formed fewer colonies than SCC9-N cells (Figure 6E-G). This result provided additional evidence that the SCC9-M cells had a lower proliferation ability than SCC9-N cells. To further investigate whether SCC9-M cells developed resistance to therapeutic drugs, mitomycin was administered to the cells for 24 h. As shown in Figure 6H, the SCC9-M cells had a higher survival rate than SCC9-N cells by treating with different drug concentrations (16 and 128 mg/ml). This result revealed that the up-regulation of MT1-MMP in SCC9 cells contributed to drug resistance of the cells. To determine the ability of resistance to apoptosis in SCC9-M cells, a flow cytometric apoptosis analysis was performed. For SCC9-M cells, the rate of apoptosis cells was lower than SCC9-N cells treated with mitomycin at the drug concentrations of both 16 and 128 mg/ml for 24 h (Figure 7A and B). Our results suggested that there existed significantly higher population of SCC9-M cells resistance to apoptosis, as shown in the statistical analysis in Figure 7C.

## Discussion

Most patients with OSCC die because of metastasis or recurrence of the tumor [2]. However, key events mediating invasion and metastasis of this carcinoma are still undefined, although the linkage between an EMT and cancer invasion and metastasis has been understood for years [5,8-10]. Studies have suggested that EMT endows cells with stem cell-like traits [26,32,33] and allows to become more invasive and migratory. Thus, our research was focused on the association of MT1-MMP, EMT and invasion and metastasis of oral carcinoma SCC9 cells; and, we made four novel observations. First, overexpression of MT1-MMP can induce oral cancer SCC9 cells to undergo EMT. Second, MT1-MMP-induced phenotypic changes in the SCC9 cells increased the level of Twist and ZEB and were dependent on repressing the transcription of E-cadherin. Third, this phenotype transformation resulted in a change in the biological properties of the cells, with the cells having decreased adhesion, high invasion but low proliferation ability. Fourth, these mesenchymal-like cells gained CSCs features.

MT1-MMP was recognized as a key mediator in both ECM remolding and cell migration during tumor progression [17,19]. Previous studies on MT1-MMP were focused on the relationship of its domain structures and cancer invasion and metastasis. Our study related to the connection of MT1-MMP and the EMT revealed that up-regulation of MT1-MMP can induce oral carcinoma SCC9 cells to undergo EMT via transcriptional repression of E-cadherin. Upon the overexpression of MT1-MMP, SCC9-M cells presented a fibroblast-like phenotype compared with the cubic epithelial phenotype of SCC9-N cells. In addition, analysis of the mRNA and protein levels verified that the SCC9-M cells underwent an EMT, in which decreased expression of epithelial markers (E-cadherin, β-catenin, cytokeratin 18) and increased expression of mesenchymal markers (vimentin, fibronectin) were observed. Furthermore, overexpression of MT1-MMP in SCC9 cells resulted in a change in the biological properties of the cells. The SCC9-M cells lost the need for cell-to-cell adhesion which contributed to cells becoming motile. As shown in invasion assay, the SCC9-M cells acquired a highly invasive ability. This in turn may allow cancer cells to cross the basement membrane and invade surrounding tissues. Importantly, recent studies demonstrated that MT1-MMP was essential for the invasive ability of cells, due to its broad-spectrum activity of degrading ECM components [16,17,34,35]. Our results verified that MT1-MMP promoted cancer cell invasion in OSCC through inducing the EMT.

The EMT is an important step in the metastatic process of epithelial tumors [10], for which recent studies have provided a more in-depth understanding of the molecular mechanisms involved. Loss of E-cadherin is central to EMT in cancer cells [5]. Thus, in the present study, we focused our attention on the transcriptional repression of E-cadherin to explain how MT1-MMP caused EMT in SCC9 cells. We also done the research to identify whether MT1-MMP overexpression resulted in the shedding of E-cadherin to induce an EMT, similar to that reported in prior studies [21,22]. However, an examination of extracellular E-cadherin in the conditioned medium on SCC9-M cells was nearly undetectable, not similarly as previously reported in prostate cancer. Our results demonstrated that MT1-MMP played a role in dynamic silencing of CDH1 so that transcriptional repression of E-cadherin, leading to the loss of the epithelial phenotype of SCC9 cells to undergo EMT. Indeed, several transcription factors that strongly repress CDH1 (such as members of Snail, ZEB and bHLH families) have recently emerged, which are now thought to be involved in tumor progression [36]. The Snail family (Snail and Slug) was first identified as inducers of EMT [37,38], and our previous work demonstrated that Snail had played an important role in inducing an EMT in SCC9 cells [39]. However, in this study, no significant difference in the expression of Snail and Slug were observed in the three experimental cells. Furthermore, the level of mRNA and protein expression observed on SCC9-M cells and SCC9-M cells treated with TIMP1 and TIMP2 may demonstrate that the MT1-MMP-induced EMT change was associated with an increase of Twist and ZEB. Twist and ZEB genes are key inducers of EMT and are closely associated with tumor progression [40-42]. However, further investigation is required, exploring the linkage between increased expression of both Twist and ZEB via MT1-MMP.

Furthermore, the more invasive SCC9-M cells did not have the ability to close the wounds in the wound healing assay. This result was consistent with our previous work [39] and revealed that the SCC9-M cells exhibited a low growth ability, which was further validated by cell-cycle analysis and cell proliferation assay. The cell mitosis of SCC9-M was blocked at the G0/G1 phase leading to a low percentage of the cells residing in S-phase, suggesting a decreased ability of cell growth. The cell growth curve showed that the PDT in SCC9-M cells was significantly longer than in SCC9-N cells, which further demonstrated this point that the SCC9-M cells displayed lower proliferation ability. Although the SCC9-M cells had lower growth ability, they possessed the ability of self-renewal, as demonstrated in the colony-forming assay. The less visible and smaller colonies formed by SCC9-M cells further elucidated that the SCC9-M cells had lower proliferation ability. It has been proposed that most of the CSCs exist in the quiescent G0 cell phase, which allows an escape from anti-cancer drug targeting and resistance to apoptosis [31]. In our study, the mesenchymal-like SCC9-M cells shared the ability of chemotherapeutic resistance to mitomycin. The flow cytometric apoptosis analysis confirmed that the SCC9-M cells are more resistant to apoptosis. These results demonstrated that there existed more SCC9-M cells in a relative quiescent state, and these non-dividing SCC9-M cells shared the ability to resistance to cell death which presented CSC-like properties. Recently, many CSC signatures have been reported, such as CD24, CD44, CD133 and so forth [4,25,43,44]. In the current study, the SCC9-M cells possessed CD24^low^ expression in contrast to the CD24^high^ expression of the SCC9-N cells, while both cell populations were CD44^high^. The expression of CD133 was either 0.89% or 0.29% in SCC9-M and SCC9-N cells, respectively. This result was not the same as that of prior reports; however, the CSC surface markers are not consistent across various tumors. The marker may or may not be useful for identifying stem cells from the other organ or tumor type [45]. Our results demonstrated that the cell surface marker CD44, while certified as a CSC marker in HNSCC, was not specific to oral SCC9 cells. Thus, it is not sufficient to define a stem cell solely based on surface markers, and multiple assays are required to isolate putative CSCs efficiently. Overall, our study demonstrated that the SCC9-M cells possessed CSC-like properties, including the ability to self-renew, resistance to chemotherapeutic agents and apoptosis, and expression of CSC markers.

## Conclusions

In conclusion, our study demonstrated that MT1-MMP, through repressing the transcription of E-cadherin, induced less aggressive oral SCC9 cells to undergo an EMT, which converted the SCC9-M cells into exhibiting a mesenchymal-like phenotype, and to possess more invasive ability. Furthermore, this transformation revealed a connection with CSCs. Collectively, further detailed information related to the molecular requirements for EMT will contribute to a better understand of tumor progression and may suggest more efficient targets for future therapeutic development.

## Abbreviations

ATCC: American Type Culture Collection; CSCs: Cancer stem cells; ECM: Extracellular matrix; EMT: Epithelial-to-mesenchymal transition; MMP: Matrix metalloproteinase; MT1-MMP: Membrane type 1-MMP; OSCC: Oral squamous cell carcinoma; TIMP: Tissue inhibitor of metalloproteinase.

## Competing interests

The authors declare that they have no competing interests.

## Authors’ contributions

CCY and LFZ carried out the experiment and performed the data analysis. XHX, TYN and JHY participated in the experiment. CCY, LKL, and LFZ designed the study, wrote and edited the manuscript. All authors read and approved the manuscript.

## Pre-publication history

The pre-publication history for this paper can be accessed here:

http://www.biomedcentral.com/1471-2407/13/171/prepub
